# Immunological subtyping of salivary gland cancer identifies histological origin-specific tumor immune microenvironment

**DOI:** 10.1038/s41698-024-00501-4

**Published:** 2024-01-20

**Authors:** Jiyun Hong, Eunwoo Choi, Dahee Kim, Mi-Kyoung Seo, Hyundeok Kang, BeumJin Park, Sangwoo Kim

**Affiliations:** 1 Department of Biomedical Systems Informatics and Brain Korea 21 PLUS Project for Medical Science, Yonsei University College of Medicine, Seoul, 03722 Republic of Korea; 2 Department of Otorhinolaryngology, Yonsei University College of Medicine, Seoul, 03722 Republic of Korea

**Keywords:** Head and neck cancer, High-throughput screening

## Abstract

Gene expression analysis enhances proper cancer subtyping, a better understanding of the molecular characteristics of cancer, and strategies for precision medicine. However, salivary gland cancer (SGC) subtyping remains largely unexplored because of its rarity and diverse histopathological and immunological characteristics. This study aimed to determine whether the histological origin and immunological characteristics of SGC subtypes are intrinsic tumor immunity factors. We performed immune profiling of 94 RNA-seq of SGC tissues and found that the SGCs that originated from the excretory duct (ED), such as the salivary duct and mucoepidermoid carcinomas, exhibit higher immunity than those from the intercalated duct (ID), such as the adenoid cystic and myoepithelial carcinomas, based on the computationally predicted immune score (*p* < 0.001), immune cell enrichment in the tumor immune microenvironment (TIME) (*p* < 0.001), T-cell receptor diversity (*p* < 0.001), and expression of signal I (major histocompatibility complex, MHC, *p* < 0.001) and signal II (co-stimulatory, *p* < 0.001 and co-inhibitory, *p* < 0.001) genes. Further analysis revealed that tolerogenic dendritic cell-induced dysfunctional T-cell populations and T-cell exclusion in the TIME are the major immune evasive mechanisms of the ED-and ID-derived SGCs, respectively.

## Introduction

Distinct molecular and clinical characteristics of different subtypes of cancers, including breast^1^, colorectal^2^, and gastric^3^ cancers can be determined by transcriptome analysis. However, salivary gland cancer (SGC) is largely unexplored owing to its low prevalence and heterogenic histopathological and genetic features. Based on the conventional classification systems by anatomical location, cell types, and molecular markers, there are currently at least 24 malignant SGC subtypes^4^. However, it is widely acknowledged that this classification may not encompass the complete spectrum of biological differences within these cancers, often leading to misclassification^5^. Moreover, considering subtype-specific genomic and transcriptomic profiling, tailored treatments optimized for each subtype are essential^6,7^.

Recent clinical trials have reported low response rates of SGCs to immune checkpoint inhibitors: confirmed objective response to pembrolizumab was 12%^8^ and the overall response rate to nivolumab was 4%^9^. Recently, studies on SGC molecular stratification are emerging, including a recent investigation of the genomic and immunological characteristics of the three major SGC types: adenoid cystic (ACC), myoepithelial (MECA), and salivary duct carcinomas (SDC) to suggest a precise immunotherapeutic strategy^10^. Currently, subtyping is underway, depending on the immunogenic and proteogenomic profiles within each subtype, with potential targets being suggested^11–13^. Similar to other cancers, such extensive studies enable an intrinsic subgrouping of SGC based on their molecular characteristics and that of their normal cell counterpart^14^ and the understanding of the molecular and morphological complexity of the salivary glands. For example, two types of reserve cells: intercalated duct (ID) and excretory duct (ED) cells can give rise to different tumors^15^.

In this study, we aimed to perform immune profiling of four major SGC subtypes: ACC, MECA, SDC, and mucoepidermoid carcinomas (MEC) using 94 RNA-seq samples from tumor tissues. Based on computational predictions of immunogenicity, active immune pathways, immune cell compositions, and the tumor immune microenvironment (TIME), supported with a comprehensive pathway-level investigation, we aimed to determine SGCs subtype-specific immunological characteristics and their association with the histological cell-of-origin involved in immune suppression mechanisms for efficient targeting off immunotherapy against SGCs.

## Results

### Transcriptomic subgrouping of the four SGC subtypes

**Fig. 1:**
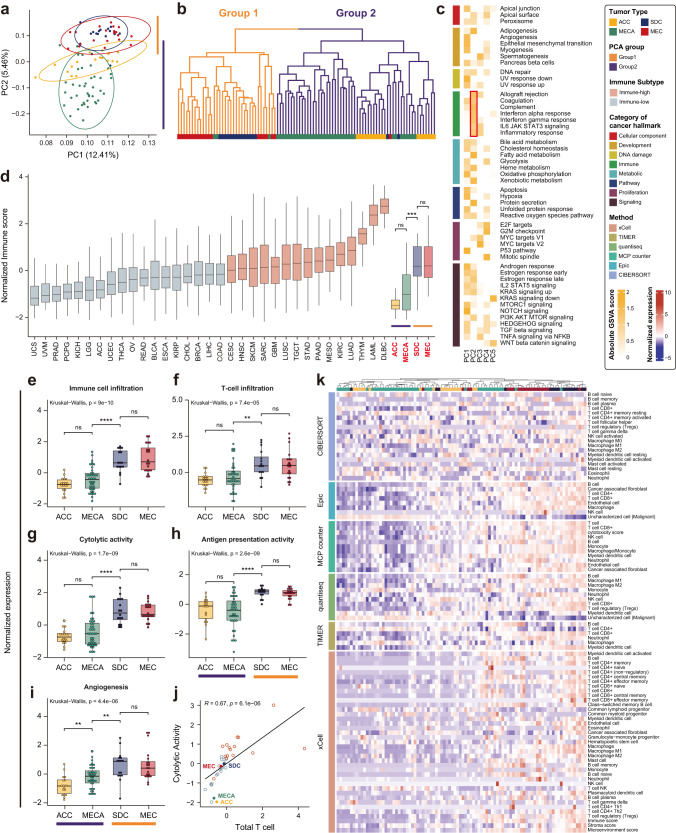
Immune landscape of salivary gland cancer (SGC) subtypes. **a** Principal component analysis (PCA) was performed on normalized expression data of the top 3000 variance genes in 94 SGC samples to identify transcriptome patterns. The percentages on the axis represent variation by components. **b** The unsupervised hierarchical clustering analysis, using normalized expression data of the top 3000 variance gene, is illustrated with a dendrogram of different colors indicating the distinct patterns of the two groups. **c** Heatmap from the single sample gene set enrichment analysis (ssGSEA) illustrating the differences in the subtypes of 50 cancer hallmark gene sets from the molecular signature database based on the first two principal components. The red box indicates consistent expression patterns of the immune categories. The normalized GSVA score represents the degree to which a gene set is up or downregulated onto PC2, which further differentiates the two groups. **d** Normalized immune score of the 31 tumor types from TCGA, including the last four boxplots, which are SGC subtypes from an independent dataset. The immune groups of TCGA cohort were classified using the 50th percentile of the immune score and its subtypes were compared using the Wilcoxon Rank Sum Test **e–i** Kruskal-Wallis Test was used to compare tumor immune microenvironment-related signatures between the subtypes. Error bars represent the standard deviation of uncertainty. **j** The scatter plot illustrates the significant positive correlation between cytolytic activity, and the total T-cell fraction based on the Pearson correlation coefficient. **k** Heatmap illustrates comprehensive immune cell composition profiling by combining six algorithms.

Transcriptomic subgrouping of the four SGC subtypes revealed distinct patterns. Through a transcriptomic analysis of 94 RNA-seq samples representing four major types of SGCs (18 ACC, 40 MECA, 16 SDC, and 20 MEC; Fig. 1), PCA-based transcriptomic similarity identified two major subgroups: SDC and MEC (Group 1) and ACC and MECA (Group 2) (Fig. 1a). Group 1 SGC subtypes largely shared an overall transcriptomic profile, whereas Group 2 SGC subtypes overlapped partially. Specifically, MECA showed larger transcriptomic variance, implying higher intrinsic heterogeneity. The two groups can be mainly differentiated by their second principal component (PC2, y-axis of Fig. 1a), whereas the first principal component (PC1, *x*-axis of Fig. 1a) was more informative for determining heterogeneity within a group. Similarly, hierarchical clustering of transcriptomic expression showed that ~92% of Group 1 was SDC and MEC, whereas ~95% of Group 2 was ACC, MECA (Fig. 1b). These results suggest the presence of a higher-level grouping pattern among the SGCs.

Gene-level mapping of the principal components (PC1 and PC2) revealed the functional factors that contributed to subgroup-specific characteristics (Fig. 1c). Among the 50 cancer hallmark gene sets of eight functional categories^16^, only seven gene signatures in the immune category (allograft rejection, coagulation, complement, interferon alpha response, interferon gamma response, IL-6/JAK/STAT3 signaling, and inflammatory response) were consistently distinguish between the two groups when mapped to PC2 (Fig. 1c) (mean GSVA score = 1.16). PCA-based clustering and immunological characteristics are major factors for SGC subtyping.

### Immune profiling of the four SGC subtypes

We calculated the overall immunity of the four SGC subtypes using computational prediction (ESTIMATE^17^) (Fig. 1d). We found that the normalized immune scores were highly distinctive among the two groups (Group 1: SDC = 0.186 and MEC = 0.2 vs. Group 2: ACC = −1.479 and MECA = −1.011; Wilcoxon Rank Sum *p*-value < 0.001). Compared to those of the 33 TCGA pan-cancer datasets, the immune scores of Group 2 subtypes were the lowest and they were classified as immune-low cancers. In contrast, the immune scores of Group 1 subtypes were in the 69th percentile, similar to that of cancers with potential immunogenicity, including lung squamous carcinoma^18,19^ and mesothelioma^19,20^ (Fig. 1d). SGC subtypes within subgroups have similar immune scores.

A detailed analysis of the TIME features confirmed the immunological differences between the two groups (Fig. 1e–i). ACC and MECA had lower immune infiltration and three T-cell activity-related (T-cell infiltration, cytolytic activity, and antigen presentation machinery) scores than SDC and MEC (Wilcoxon Rank Sum *p*-value < 0.0001); however, the scores were not different within the subgroups (Fig. 1e–h). Only the angiogenesis score further differentiated between ACC and MECA (Fig. 1i). Moreover, correlation analysis showed that T-cell population and cytolytic activity are positively correlated and distinct for each group (Fig. 1j), indicating that T-cell-mediated immunity contributes to the immunological differences between the groups.

We further examined individual cell type TIME landscape profiles using six different algorithms: xCell^21^, TIMER^22^, quantiseq^23^, MCP-counter^24^, Epic^25^, and CIBERSORT^26^ (Fig. 1k). We observed an overall increase in the number of cells involved in both innate and adaptive immunity against SDC and MEC, including cytotoxic T-cells (CD8 + ), activated NK cells, B-cells, and macrophages. In contrast, uncharacterized cells, which were considered malignant cells, and common lymphoid/myeloid progenitors showed the opposite or no correlation. These results confirmed the immune-low and immune-high features of Groups 1 and 2 subtypes, respectively, regarding both innate and adaptive immunity.

### Association between histological origin and immunogenicity of SGC subtypes

**Fig. 2:**
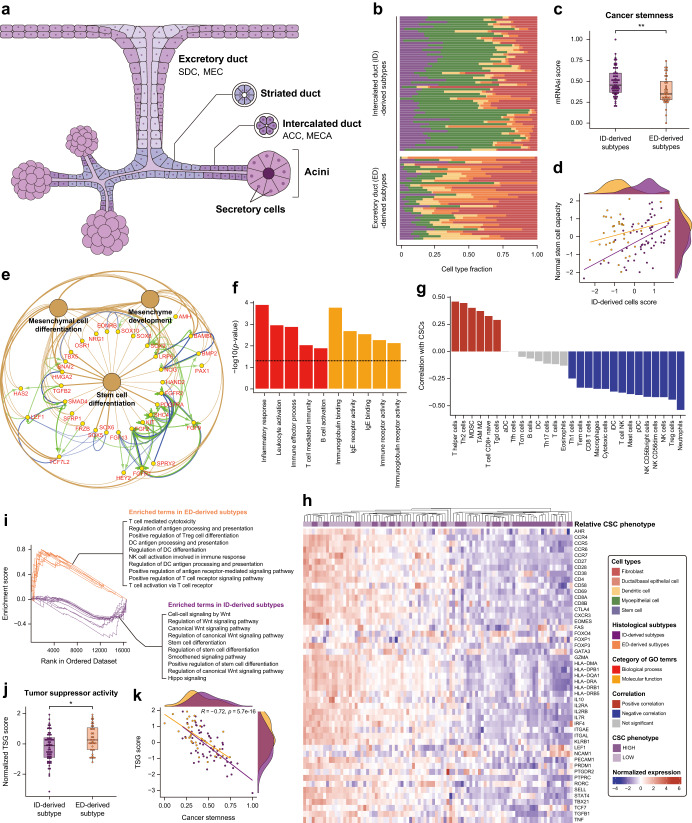
Histological origin of the salivary gland cancer (SGC) subtypes. **a** Diagram illustrates the four ductal units of the human salivary gland where SGC can occur: acinar (AC), intercalated duct (ID), striated duct (SD), and excretory duct (ED). The salivary gland consists of three ductal units: intercalated, intralobular (striated), and interlobular (excretory) ducts. **b** Cell type decomposition analysis compared SGC subtypes originating from the ID and ED, with different cell types indicated by various colors. **c** Comparison of cancer stemness between the two groups. **d** Scatter plot illustrating a moderately positive association between ID cells, considered as stem cells in the salivary glands, and the stemness of SGC subtypes originating from the ID. **e** Gene network analysis including up-regulated Differentially Expressed Genes (DEGs) associated with Gene Ontology (GO) terms related to mesenchymal stem cells in ID-derived subtypes. Brown nodes and edges represent significant GO terms and their interactions. Genes associated with these terms are indicated by yellow color within the circles. Green edges and arrows signify activation mechanisms, while blue edges and arrows represent binding interactions. **f** GO terms related to the immune system were identified through downregulated Differentially Expressed Genes (DEGs) in the higher cancer stemness group compared to the lower group. **g** Histogram illustrates the correlation (positive or negative) between the immune cells and CSCs. The red and blue colors represent the correlation coefficient, and the gray color indicates a multiple testing corrected *p*-value > 0.05. **h** The heatmap displays a comparison of T-cell activation marker expression in the higher CSC group compared to the lower CSC group. **i** GSEA result indicating enriched terms by histologic subtypes: ID-derived subtypes showed many oncogenic signaling pathways and regulation of stem cell-related gene sets, whereas ED-derived subtypes exhibited immunogenic cell types and regulation of antigen processing and presentation mechanisms. **j** comparison of tumor suppressor function between the two groups. **k** A strong negative correlation between cancer stemness and tumor suppressor gene score.

We examined the association between the histological origin and the immunogenicity of the identified subtypes. Previous studies have shown that SGC originated from two of the four major substructures in the salivary gland (Fig. 2a): ACC and MECA are intercalated duct (ID)-derived and SDC and MEC are excretory duct (ED)-derived^27–29^. Cell type decomposition analysis confirmed that the samples used in this study had similar origins (Fig. 2b). In addition, we found that the ACC and MECA samples had a higher proportion of stem cells and myoepithelial cells (Wilcoxon Rank Sum *p*-value < 0.001), whereas the SDC and MEC samples exhibited abundant fibroblasts, dendritic cells (DCs), and ductal/basal epithelial cells (Wilcoxon Rank Sum *p*-value < 0.001). These results are consistent with the known cellular compositions of ID (higher rate of cellular division and stem cell properties) and ED (higher number of professional antigen-presenting cells (APCs))^30–34^.

We further analyzed whether histological traits could be the source of the different immunogenicity of the SGCs. The computationally predicted score^35^ showed higher cancer stemness in the ID-derived subtypes than in the ED-derived subtypes (Fig. 2c). Moreover, we observed a positive correlation (*R* = 0.5, *p* < 0.001) between intercalated duct cells, which are known to exhibit stem cell properties in salivary glands and potential stemness to differentiate into various glandular cell types, particularly ID-derived subtypes (Fig. 2d).

Additionally, our gene network analysis associated with GO terms related to mesenchymal stem cells revealed a decrease of MHC molecules in ID-derived subtypes (Fig. 2e and Supplementary Fig. 4). Furthermore, we found that the higher cancer stem cell (CSC) group, regardless of histological origin, exhibited downregulated immune system activity (Fig. 2f). Cell type associations also indicated that CSC fractions correlated negatively with the majority of activating immune cells, including CD8 + T, cytotoxic, and NK T-cells, and positively with suppressive immune cells, including Th2 cells, myeloid-derived suppressor cells, tumor-associated macrophages, and naïve CD8 + T-cells (Fig. 2g). Specifically, gene expression of all types of T-cell activation markers was largely suppressed in the higher CSC group, composed mainly of ID-derived subtypes (~75%, Fig. 2h). This highlights that immune system activity decreased in the group with a higher abundance of cancer stem cells, irrespective of histological origin. These findings suggest that the higher proportion of stem cells in ID-derived cancer contributed to the abundance of CSC during tumorigenesis, promoting immune escape through myeloid progenitor-derived cells^36,37^.

Pathway-level analysis revealed the potential underlying mechanisms of ID-derived cancer cell stemness (Fig. 2i). GSEA showed the enrichment status of the Wnt (Combined FDR = 1.2e-08), Hippo (4.3e-02), and Smoothened (2.4e-02) oncogenic signaling pathways, along with the regulation of stem cell differentiation (Combined FDR = 1.5e-04) in ID-derived subtypes. Enrichment analysis of up-regulated DEGs in ID-derived subtypes also identified cAMP (Combined FDR = 0.032), Rap1 (0.024), TGFβ (0.153e-11), Hippo (0.68e-11), and Wnt signaling pathways (0.26e-24) (Supplementary Fig. 3). Specifically, we suggested that the SMAD complex (SMAD2/3/4) positively interacts with OCT4 (POU5F1), one of the essential markers of cancer stemness, as indicated by network analysis. In addition, a lower tumor suppressor gene (TSG) score in the ID-derived subtypes (Fig. 2j) and its strong negative correlation with cancer stemness (Fig. 2k) suggested that TSG alleviation may contribute to the proliferation of CSCs as reported in a previous study^38^. Overall, our analysis showed that distinct immunological traits were affected by the histological origin of the tumors, primarily differentiating SGCs into ID- and ED-derived subtypes.

### Prediction of subtype-specific immune evasion mechanisms of SGC

**Fig. 3:**
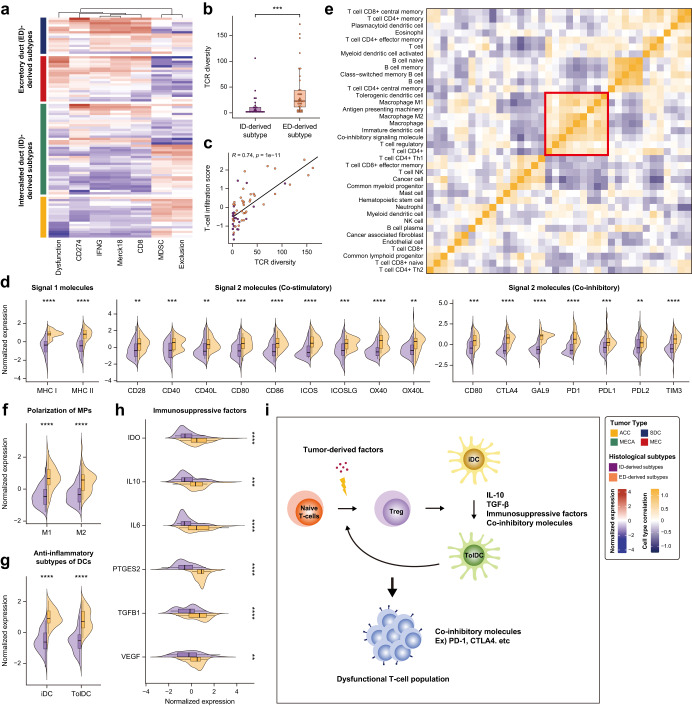
Mechanisms underlying dysfunctional T-cell populations in ED-derived SGC subtypes. **a** Heatmap of T-cell functionality against SGC subtypes, visualized using ssGSEA with the tumor immune dysfunction and exclusion (TIDE) signatures. Expression of TIDE features significantly differs between the groups (*p* < 0.05). **b** Comparison of estimated T-cell receptor (TCR) diversity using CDR3 sequence between the two groups. **c** Scatter plot illustrating a positive correlation between median T-cell infiltration and TCR diversity. **d** Comparison of signaling molecules between the two groups. The statistical significance was confirmed using the Wilcoxon Rank Sum Test. **e** Correlogram depicting the correlation between immune cell types. Colors indicate the strength of the correlation (blue – negative correlation, white – no correlation, yellow – positive correlation) and the red box highlights the malfunction of professional APCs. Comparison of (**f**) macrophage polarization (**g**) dendritic cell subtypes, and (**h**) immune suppressive molecules between the two groups. **i** Hypothetical model illustrating the formation of dysfunctional T-cell populations in ED-derived subtypes.

To identify subtype-specific T-cell functionality, we performed a gene set analysis based on TIDE (Fig. 3). CD8 and Merck18 T-cell inflamed signatures, interferon-gamma (a pro-inflammatory molecule), and dysfunctional T-cell signatures (CD274 and T-cell dysfunction scores) increased in the ED-derived subtypes (Fig. 3a). In contrast, the ID-derived subtypes showed increased myeloid-derived suppressor cells which were associated with T-cell exclusion (Fig. 3a). Moreover, the T-cell exclusion score was higher in ID-derived subtypes. Additionally, mixed T-cell functionality was observed in MECA. Overall, these findings provide important insights into tumor immune dysfunction and exclusion mechanisms in different histological subtypes, with ED- and ID-derived subtypes associated with increased dysfunctional T-cell population and excluded T-cell population, respectively.

Moreover, we analyzed the T-cell receptor beta-chain-CDR3 sequences to investigate the ability of T-cells to recognize antigens in the two groups (Fig. 3b) and found that the ED-derived subtypes had higher TCR diversity, which was positively correlated with the T-cell infiltration score (Fig. 3c). These findings suggest that the ED-derived subtypes exhibited increased sensitivity to antigens and more active T-cell infiltration, indicating that dysfunctional T-cells were not caused by low TCR diversity. In contrast, the ID-derived subtypes exhibited low TCR diversity in the excluded T-cell population, indicating a lack of T-cell functionality.

To explore the underlying mechanisms of the observed differences in T-cell functionality between the ED- and ID-derived subtypes, we further analyzed the expression of molecules involved in antigen presentation and recognition and the co-inhibitory molecules that suppressed T-cells (Fig. 3d) and found that ED-derived subtypes had a higher expression of signal 1 molecules, including MHC I, MHC II, and TCR, and signal 2 co-stimulatory molecules, such as CD80/CD86, CD40, OX40L, ICOSL, CD28, CD40L, OX40, and ICOS. T-cell-suppressing co-inhibitory molecules were also elevated in ED-derived subtypes. The high expression of co-inhibitory molecules in the ED-derived subtypes, including GAL9, TIM3, CTLA4, CD80, PD-L1, and PD-1, was consistent with the results shown in Fig. 3a (for example, CD274). These findings suggest that co-inhibitory molecules may influence dysfunctional T-cell mechanisms, and that weak signal 1 and co-stimulatory molecules alone may be insufficient to activate the T-cell priming process in ID-derived subtypes. This could result in the exclusion of T-cell populations with low tumor specificity.

Furthermore, we observed a correlation between immune cells, suggesting a malfunction in antigen processing and the presentation mechanism of professional APCs (Fig. 3e). This led us to investigate the specific cell types that may contribute to the observed elevation of co-inhibitory molecules and dysfunctional T-cells in ED-derived subtypes despite the high expression of signal 1 and co-stimulatory molecules.

We found that macrophages and DCs are professional APCs that formed cluster based on their antigen-presenting machinery scores and similar phenotypes, including tolerogenic and immature DCs, M1- and M2- macrophages (red boxes). Additionally, our analysis revealed that ED-derived subtypes were associated with a suppressive phenotype of professional APC and that the anti-inflammatory subtypes of macrophages (M2-macrophages) and DCs (immature and tolerogenic [tolDCs]) were strongly associated with immunosuppressive factors, such as co-inhibitory signaling molecules and regulatory T-cells.

Both pro-inflammatory M1-^39^ and anti-inflammatory M2-polarized macrophages^40^ were elevated in the ED-derived subtypes; however, the ratio of M2 to M1 in the ID-derived subtypes was higher (Fig. 3f). This suggests that ID-derived subtypes were primarily affected by the anti-inflammatory phenotype of macrophages compared to ED-derived subtypes. Additionally, DCs were another type of professional APC that maintain the balance between adaptive immunity and the tolerogenic response to tumors during antigen processing and presentation. Our results showed that there were higher numbers of mature and activated DCs and immature and tolDCs in the ED-derived subtypes than in the ID-derived subtypes (Fig. 3g).

Tregs and tolDCs are crucial in creating an environment that induces immunosuppression. It has been reported that Tregs inhibit the DCs co-stimulatory signaling molecules and inhibit DC-mediated pro-inflammatory cytokines, IL-12, and TNF*-a*^41^. In contrast, Tregs upregulate anti-inflammatory cytokines TGF-β and IL-10, which have tolerogenic properties^42,43^. Anti-inflammatory factors (TGF-β, IL-10, IL-6, VEGF, IDO, and PGE2), which contributed to immunosuppressive environment were highly expressed in ED-derived subtypes than in ID-derived subtypes (Fig. 3h).

Summarily, we presented a hypothetical model that explained the malfunctioning of professional APCs and the formation of dysfunctional T-cell populations in ED-derived subtypes. Tumor-derived factors inhibit the differentiation and maturation of DCs and promote the accumulation of iDCs^41,44^. Continuous stimulation of naïve T-cells by increased levels of iDCs and tumor-derived factors leads to the activation of Tregs, which subsequently promotes the differentiation of iDCs into tolDCs. This creates an environment that increases co-inhibitory signaling molecules, facilitated by the ability of iDCs to secrete IL-10 and many other immunosuppressive factors. Furthermore, there is a bidirectional relationship between tolDCs and Tregs: Tregs affect the activation of tolDCs and activated tolDCs can increase Tregs through positive feedback loops^41^ as illustrated in Fig. 3i.

### Validation with independent datasets and Immunohistochemistry (IHC) analysis

**Fig. 4:**
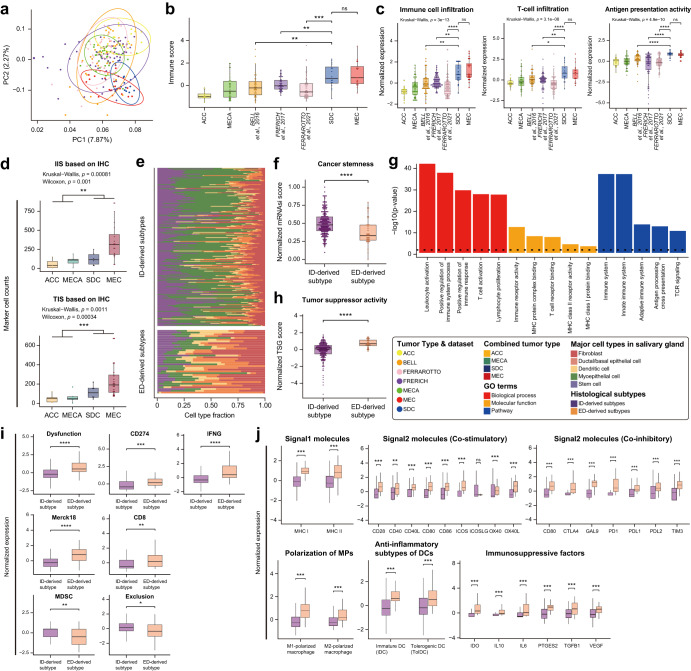
IHC and in-silico validation with independent datasets. **a** PCA-based clustering ACC, MECA, and three independents, along with SDC and MEC, based on normalized expression. **b** Comparison of overall immune scores in the tumor microenvironment among histological subtypes using computational estimates. **c** Comparison of all TIME features illustrating differences in immunity among subtypes. **d** Immunohistochemistry (IHC) targeting infiltrating T-cells and immune cells labeled with CD3/CD45. **e** Comparison of significantly different cell type compositions by origin-derived subtypes. Comparison of (**f**) cancer stemness and (**h**) tumor suppressor activity. **g** Enriched GO terms in ID-derived subtypes using downregulated DEGs compared to ED-derived subtypes. Comparison of (**i**) tumor immune-related scores and molecules, specifically focusing on T-cell dysfunction, MDSC, and T-cell exclusion, as well as (**j**) immune signaling molecules, immunosuppressive factors, and subtypes of antigen-presenting cells.

To validate the transcriptomic profiles based on the intrinsic cell composition of histologic origins, we incorporated three available independent datasets (42 ACC; Bell et al.^45^, 58 ACC; Frerich et al.^46^, and 54 ACC; Ferrarotto et al.^11^) and attempted to reproduce our findings (Fig. 4). The transcriptomic subgrouping revealed two PCA-based groups. ACC, MECA, and the three public ACC datasets (upper panel) and SDC and MEC (lower panel) were characterized by the second principal component (Fig. 4a). Consistent with the previous finding (Fig. 1a), the independent datasets clustered around ACC and MECA, indicating coherence between ACC and MECA subtypes. This suggests that the expression profiles of ACC were similar to MECA, while differing from SDC and MEC. Likewise, we computationally estimated overall immunity and compared to validate the immune profile of histological subtypes (ACCs = −0.14 and MECA = −0.25 vs. SDC = 0.83 and MECA = 1.02; Wilcoxon Rank Sum *p*-value < 0.001), reproducing ED-derived subtypes have higher immunity than ID-derived subtypes (Fig. 4b). Morris et al.‘s ACC exhibited statistically significant lower immunity compared to ACCs in Bell et al, Frerich et al, and Ferrarotto et al*.*‘s dataset (all three Wilcoxon Rank Sum *p*-value < 0.01). However, the difference in immunity between Morris’s ACC and MECA was statistically significant only in Frerich’s dataset (Wilcoxon Rank Sum *p*-value < 0.01), indicating that Morris’s ACC was significantly lower than other ACCs but did not show a significant difference compared to MECA.

Additionally, our high-resolution TIME analysis provided further support for the differences in overall immunity, revealing consistent patterns where ED-derived subtypes exhibited higher scores in all features (Wilcoxon Rank Sum *p*-values < 0.01, Fig. 4c and Supplementary Fig. 5). We performed CD3/CD45 IHC on all MEC patients to validate TIS/IIS and compared to ACC, SDC, MECA from Morris’s dataset, presenting consistent gradual pattern with computational estimates (Fig. 4d and Supplementary Fig. 6). Furthermore, we performed a deconvolution of the cell composition within the Tumor Immune Microenvironment (TIME) and conducted unsupervised clustering based on all cell types (Supplementary Fig. 7). Although the clustering did not align with the histological subtypes we previously explored (Fig. 1k), the ED-derived subtypes exhibited higher expression levels in both innate and adaptive immune cells and had lower malignant scores compared to the ID-derived subtypes. Not only among immune cells but also among the abundant cell types within the salivary gland, our comparison revealed that in the histologic intrinsic environments, stem cells and myoepithelial cells predominate in the ID-derived subtypes (Wilcoxon Rank Sum *p*-value < 0.001), whereas fibroblasts, ductal/basal epithelial cells, and dendritic cells were more abundant in the ED-derived subtypes (Wilcoxon Rank Sum *p*-value < 0.001).

Moreover, the analysis of cancer stemness and tumor suppressor activity had been further strengthened and clarified by the addition of independent datasets (Fig. 4f, h). We reproduced that cancer stemness score was higher in the ID-derived subtypes and had lower score than ED-derived subtypes. Their correlation might be the potential evidence regarding the effect of cancer stemness on tumor suppressor function regardless of histologic origin (Supplementary Fig. 8). Next, enriched GO term analysis represented alleviated T-cell immunity and antigen processing and presentation related molecules in the ID-derived subtypes (Fig. 4g). In fact, tumor immune-related molecules highly elevated in the ED-derived subtypes, specially T-cell dysfunction score was upregulated in the ED-derived subtypes while MDSC and T-cell exclusion score was downregulated in the ID-derived subtypes (Fig. 4i). Additionally, we validated the immune signaling molecules, immunosuppressive factors, and subtypes of APC related to crosstalk between the histological intrinsic characteristics and immune-escape mechanisms (Fig. 4j). All features in the independent datasets were consistent with our previous findings, supporting the presence of abundant CSCs with rapid cell division and relatively low immunogenicity in ID-derived subtypes, while ED-derived subtypes exhibited higher APCs along with immunosuppressive factors, forming a dysfunctional TME.

## Discussion

In this study, we aimed to identify histologically related SGC subtypes based on multifaceted TIME and immunogenicity analyses. We showed that the four major SGC subtypes can be subdivided into ID- and ED-derived subtypes that exhibit immune-low and -high features, respectively. The molecular and histological characteristics of normal cells (high stemness in ID and high number of APCs in ED) were closely associated with their oncogenic counterparts. Finally, proliferative cancer stemness, T-cell exclusion, and APC malfunction were predicted to be the major causes of immune evasion in ID- and ED-derived subtypes and could be effective targets for cancer immunotherapy.

Despite recent studies suggesting that the specific features of the SGC subtype could be potential immunotherapeutic targets, there are currently no optimal therapies for SGC. This highlights the need for further research to enhance our understanding of the molecular and genetic characteristics of SGC subtypes and develop effective precision medicine strategies for each subtype. Unlike previous studies that mainly focused on identifying subtypes based on the immune microenvironment, our study presents a more integrated and comprehensive perspective of SGC subtypes.

According to the PCA-level mapping pathway analysis, we observed that Group 1 and Group 2 were distinguished primarily by PC2, which demonstrated a strong association with the immunogenic pattern. This observation further underscores the crucial role that immunogenicity plays in differentiating the SGC subtypes. Conversely, PC1 did not elucidate the differences between the groups, nor did it exhibit a clear transcriptional expression pattern. Rather, PC1 reflected the heterogeneity inherent within each group. So, we focused on the immune groups driven by PC2, and embarked on an investigation into the factors that might influence the immunogenicity within the TME at the cell type level.

We employed six cell deconvolution algorithms together with a signature gene set to examine a variety of factors within the TME. Despite certain algorithms eliciting imprecisions in the quantification at the cell type level for designated markers, including T-cell CD4 + , common myeloid progenitor, and T-cell CD4 + Th1, we effectively leveraged comprehensive measurements to encompass a broad array of cell types, thereby substantiating more robust results. While we acknowledged minor inconsistencies in both cell type markers and principles across the different algorithms, we remain highly confident that the consensus results, which showed enrichment in almost all immune cells, were predominantly observed in Group 2. Furthermore, we investigated the potential capacity of T-cells to respond to immunotherapy. Intriguingly, despite the escalation in tumor immunity-related signatures and TCR diversity within Group 2, we concurrently discerned an amplified incidence of specific immune suppressive factors and inhibitory molecules linked with dysfunctional T-cells within this group. This insinuates that these suppressive cells could potentially modulate the immune escape mechanisms.

Considering the distinction between the immune desert and the dysfunctional TME, despite their similar subtypes, we integrated histological, immunological, and molecular features to yield a more comprehensive understanding of the driving mechanisms behind each subtype. This insight could potentially facilitate the development of effective and precise therapeutical strategies for SGC. Specifically, ID-derived subtypes show high stemness features, whereas ED-derived subtypes show high levels of APCs. Differences in normal cell-origin characteristics were observed in their oncogenic counterparts.

Based on these findings, different precise treatment strategies can be proposed for each subtype. For example, in ID-derived subtypes, where cancer stems cell proliferation and oncogenic signaling pathways have been identified as the main causes, effective treatment strategies that eliminate cancer stem cells and suppress their proliferation are required. Possible treatments include cell cycle inhibitors or agents targeting CSC features^47^. Conversely, in the ED-derived subtypes, where T-cell and APC dysfunction has been identified as the main causes, treatment strategies are needed to restore T-cell function through immune checkpoint blockade or chimeric antigen receptor-T-cell therapies^48^.

Similarly, recent studies have demonstrated further subtyping within both ACC and MEC based on immunogenic and proteogenomic profiling within each subtype^11–13^. ACC has been classified into immune-hot and -cold subtypes, with immune checkpoint B7-H4 identified as a therapeutic marker for the immune-cold subtype. In contrast, MEC has been categorized into immune subgroups based on immune cell status. Previous research has indicated that these immune subgroups can induce heterogeneity within histologic subtypes. According to our results, it is crucial to consider additional factors, such as immune checkpoint molecules, especially in samples exhibiting diverse intrinsic cell compositions based on histologic origin.

We observed heterogeneity in the scores while comparing immunity between the histological subtypes (Fig. 3d–h). Challenges in obtaining specific clinical features from public datasets have limited our ability to identify factors responsible for this heterogeneity, such as diagnosis, demographics, or treatment. Further analyses incorporating detailed clinical information are necessary to understand the influencing factors within these subtypes.

Additionally, we explored the impact of PCA-based sample outlier and immune subtypes on this heterogeneity. Firstly, we considered outliers based on gene expression profiles deviating from SGC subtypes and removed them. Consequently, we obtained clearer statistical significance between origin-derived subtypes (Supplementary Fig. 1). Secondly, we identified distinct immune subtypes within the same subtype. In line with previous studies^12,13^, significant differences were observed between immune subtypes in each subtype (Supplementary Fig. 2a–f). Classifying immune subtypes within the same subtype could enhance our understanding of heterogeneous events in origin-based subtypes.

Consistent with the results of previous studies^10^, we found that MECA exhibits heterogeneous T-cell functionality. Specifically, half of the MECA samples showed characteristics similar to those of ED-derived subtypes. To further investigate the cellular composition of MECA, we compared ID-like and ED-like MECA samples. No significant differences were observed between the two subtypes regarding the level of fibroblast, ductal/basal epithelial cells, and intercalated duct specific cells (myoepithelial cells). However, ED-like MECA had higher numbers of macrophages and DCs in the excretory duct, which consist of excretory duct. Although MECA can be classified as an ID-derived subtypes based on its histological origin, MECA subtypes with heterogeneous cellular composition, including high APCs resembles ED-derived subtypes. Therefore, combination therapies that target both ID and ED subtypes may be more effective for MECA, which exhibit characteristics of both subtypes.

Despite comprehensive and in-depth analyses, our study has a few limitations. First, the number of bulk RNA-seq samples used was relatively few (*n* = 94). Single-cell transcriptomics increases the resolution of cell composition and cell-type-specific pathway status identification. We hope that the rarity and lack of available samples will be resolved in the near future to ensure the increased availability of fresh tumors and normal tissues for single-cell analysis. Second, different types of genome-scale data, such as genomic mutations and epigenetic alterations, will provide more opportunities for multifaceted analyses, such as tumor mutation burden or neoantigen burden analysis and the regulatory mechanisms of the identified pathways. MYC-NFIB fusion gene is prevalent in > 70% of ACC cases and is a major driver of this malignancy^49^. The role of the fusion gene in immune suppression can be assessed using genomic data (such as fusion gene-derived neoantigens) and downstream transcriptome effects. Finally, our study was primarily based on computational analysis, which requires further confirmatory tests. We hope that a more active collection of tumor tissues or the construction of patient-derived models (such as patient-derived xenograft) will expedite the active validation and discovery of resistance mechanisms against conventional immunotherapy.

In conclusion, our study suggests that the cellular composition of normal cells may play a role in determining the characteristics of their oncogenic subtypes. Our findings highlight the relationship between histological origin and the TIME, which can be used to propose potential mechanisms of cellular components that contribute to tumorigenesis.

## Methods

### Sequencing data and sample acquisition

Data from 76 cases (20 adenoid cystic carcinomas, 40 myoepithelial carcinomas, and 16 salivary duct carcinomas) used in the Memorial Sloan Kettering Cancer Center study^10^ were collected from the National Center for Biotechnology Information Sequence Read Archive (SRA) (https://www.ncbi.nlm.nih.gov/sra). FASTQ files were downloaded with the “*fasterq-dump*” function of the SRA Toolkit. Additionally, we included twenty cases of mucoepidermoid carcinomas (MEC) from our previous study^13^. In accordance with the Declaration of Helsinki, we obtained informed written consents from all MEC patients, which were approved by the Institutional Review Board at Severance Hospital, Yonsei University College of Medicine (IRB 255-001). The specific criteria for selecting MEC patients were clearly defined in the previous description and are detailed in Supplementary Table 1. In this study, primary tumors and corresponding normal tissues from twenty patients with mucoepidermoid carcinoma were utilized. Fresh frozen tissues obtained during surgery were processed for RNA sequencing library preparation using the TruSeq Stranded Total RNA Sample Prep Kit protocol. The quality of the libraries, including the integrity of total RNA, was assessed using the Agilent 2100 BioAnalyzer. Subsequent sequencing was conducted using the Illumina HiSeq 2500 system.

### RNA-seq processing and analysis

FASTQ files with adapter sequences and low-quality reads were checked using FastQC v0.11.9 and processed using Trimmomatic v0.40 with the following options: illuminance:2:30:10, LEADING:20, TRAILING:20, MIMLEN:20, and CROP:72. The remaining reads were then aligned to the human reference genome (GRCh38) using STAR v.2.7.3a in two-pass mapping mode. The read counts for each sample were obtained using HTSeq v0.11.1. To reduce bias caused by low expression, genes with more than half of the samples in each subtype having a zero count or an average count of < 10 in the group were excluded. Subsequently, relative gene expression was normalized using the “vst” function of the DESeq2 R package v1.26.0^50^. Combat-seq^51^ and SVA were used to identify non-biological batch effects and hidden sources of data variation.

### Gene expression profiling with highly variable genes

We performed principal component analysis (PCA) to identify the highest biological variation using the normalized gene expression profiles of the top 3000 genes. PCA was conducted using the “*prcomp*” function in base R. Unsupervised hierarchical clustering analysis (HCA) based on similar gene expression patterns was conducted using the stats R package. We measured the Euclidean distance between two groups and clustered them using the complete method. Based on the PCA results, the principal components were mapped onto 50 cancer hallmark gene sets using single-sample gene set enrichment analysis (ssGSEA). GSEA was performed using a normalized gene expression matrix with the gene set variation analysis (GSVA) R package v1.44.5 and clusterProfiler R package v3.14.3. As a reference, gmt files were downloaded using the msigdbr R package v7.5.1 (https://github.com/igordot/msigdbr).

### The cancer genome atlas (TCGA) data acquisition and analysis

Public RNA-seq read count data were downloaded from TCGA to compare the immunity of pan-cancers with salivary gland carcinomas. We collected the HTSeq-count based expression data of pan-cancers (33 cancer types) using TCGAbiolinks R package v2.21.3: uterine carcinosarcoma (*n* = 56), prostate adenocarcinoma (*n* = 551), pheochromocytoma and paraganglioma (*n* = 186), uveal melanoma (*n* = 80), kidney chromophobe (*n* = 89), brain lower grade glioma (*n* = 529), adrenocortical carcinoma (*n* = 79), uterine corpus endometrial carcinoma (*n* = 587), ovarian serous cystadenocarcinoma (*n* = 379), thyroid carcinoma (*n* = 568), rectum adenocarcinoma (*n* = 177), esophageal carcinoma (*n* = 173), kidney renal papillary cell carcinoma (*n* = 321), cholangiocarcinoma (*n* = 45), liver hepatocellular carcinoma (*n* = 424), colon adenocarcinoma (*n* = 521), bladder urothelial carcinoma (*n* = 433), breast invasive carcinoma (*n* = 1222), glioblastoma multiforme (*n* = 174), cervical squamous cell carcinoma and endocervical adenocarcinoma (*n* = 309), head and neck squamous cell carcinoma (*n* = 546), skin cutaneous melanoma (*n* = 472), sarcoma (*n* = 265), stomach adenocarcinoma (*n* = 407), testicular germ cell tumors (*n* = 156), lung squamous cell carcinoma (*n* = 551), pancreatic adenocarcinoma (*n* = 182), mesothelioma (*n* = 86), kidney renal clear cell carcinoma (*n* = 611), lung adenocarcinoma (*n* = 594), thymoma (*n* = 121), acute myeloid leukemia (*n* = 151), and lymphoid neoplasm diffuse large b-cell lymphoma (*n* = 48).

### Profiling of tumor immune microenvironments (TIME)

Stromal and immune cell levels were determined using the ESTIMATE algorithm^17^ and the immune score was considered the total immunity in the TIME. The pan-cancer immune subgroups were classified using the 50th percentile. Furthermore, immune cell population gene signatures were used to calculate the immune infiltration, T-cell infiltration, antigen-presenting machinery, and angiogenesis scores with ssGSEA as described previously^52^. Cytolytic score (CYT) was calculated as the geometric mean expression of GZMA and PRF1 (CYT score = √ GZMA × PRF1), as described previously^53^. We performed a correlation analysis between the total T-cell fraction and cytolytic activity, which was considered significant if (1) *P* < 0.05 and (2) correlation > 0.2 (up or downregulated). Similarly, we performed the deconvolution of cell-type fractions using immunoedeconv R package v2.1.0 by incorporating six algorithms (xCell^21^, TIMER^22^, quantiseq^23^, MCP counter^24^, Epic^25^, and CIBERSORT absolute mode^26^). T-cell functionality, including dysfunction and exclusion scores, was inferred from the Tumor Immune Dysfunction and Exclusion (TIDE)^54^. We considered samples that were smaller than (Q1 - 1.5 * interquartile range (IQR)) or larger than (Q3 + 1.5 * IQR) as outliers and removed them. Markers for measuring immune cell type, T-cell activation, and TIME activity used in this study are described in Supplementary Table 2.

### T-cell receptor (TCR) repertoire analysis

The TCR repertoire was analyzed using MIXCR v3.0.13^55^ with paired-end reads, which were aligned using the RNA-seq mode. Aligned reads were assembled to extract TRB-CDR3 gene regions using default parameters based on the developer instructions (https://github.com/milaboratory/mixcr/). As TCR clonotypes cannot be effectively detected below the 100 bp read length, TCR analysis was performed only with sixty-eight samples with a 200 bp read length. The remaining samples were further analyzed and visualized using the Immunarch R package v0.6.6.

### Stemness & tumor suppressor gene analysis

Cancer cell stemness was measured using an mRNA expression-based stemness index (mRNAsi)^35^. To calculate mRNAsi, we downloaded the Progenitor Cell Biology Consortium and TCGA PanCanAtlas datasets and trained stemness signatures using normal stem cells following the workflow proposed by PanCanStem (https://github.com/ArtemSokolov/PanCanStem). A one-class logistic regression algorithm was used to estimate the stemness index for each sample. Normal stem cell capacity and tumor suppressor gene score were estimated using single-sample gene set enrichment analysis (ssGSEA) with all the human suppressor genes (Supplementary Table 2).

### Cell type decomposition analysis

The cellular composition was inferred using the BayesPrism R package v2.0^56^. We trained the model using public single-cell RNA-seq data for adenoid cystic carcinoma (GSE210171) as prior information and subsequently, performed deconvolution. The decomposed cell types were annotated using ScType^57^. The annotated cell types were used as reference cells for downstream analyses (Supplementary Table 4).

### Gene network analysis

We input differentially expressed genes (DEGs) that were up- and downregulated between the ID- and ED-derived subtypes. Subsequently, we performed an analysis of Gene Ontology (GO) terms and pathways, utilizing the REACTOME database. To illustrate their interactions, we selected specific terms and genes associated with cancer stemness and potential signaling pathways. These analyses were deemed significant under a *p*-value threshold of 0.05, employing ClueGO and CluePedia within the Cytoscape software.

### Immunohistochemistry

Formalin-fixed paraffin-embedded (FFPE) tissue blocks of MEC tumors that were previously sequenced, were sectioned onto glass slides at 5-um. Hematoxylin and eosin (H&E) stains were performed following standard procedures. Anti-CD45 (DAKO, catalog no. M0701, 0.5 mg/mL) and anti-CD3 (DAKO, catalog no. A0452, 1.2 mg/mL) antibodies were applied and sections were incubated for 24 h, followed by a 60-min incubation with biotinylated horse anti-mouse IgG (Vector Laboratories, catalog no. PK-6200) for CD45 antibody, and horse anti-rabbit IgG (Vector Laboratories, catalog no. PK-6200) for CD3. Slides were counterstained with hematoxylin and enclosed in a coverslip with Permount cover medium (Thermo Fisher Scientific). Slides were digitally scanned, and the quantification of stained slides was performed by counting the number of positive leukocytes in the central region of the tumor under 400x magnification (Supplementary Table 3). The fields were chosen randomly by a head and neck/salivary pathologist blinded to the immune infiltration score (IIS) and T-cell infiltration score (TIS) values, in the central region of the tumor. In total, positively stained leukocytes were counted digitally in 3 fields per section via QuPath 0.4.4 (https://qupath.github.io.) and the arithmetic mean was used for statistics.

### Statistical test

We employed statistical test to evaluate differences between groups and determine significance. Throughout this study, group comparisons were conducted using the Wilcoxon Rank Sum test. For multiple group comparison, the Kruskal-Wallis test was used. To account for multiple comparisons, corrections were made using both the False Discovery Rate (FDR) and Bonferroni correction methods. Statistical significance was evaluated between groups according to following threshold: *P < 0.05, **P < 0.01, ***P < 0.001, ****P < 0.0001.

### Reporting summary

Further information on research design is available in the Nature Research Reporting Summary linked to this article.

### Supplementary information

REPORTING SUMMARY

supplementary information - final submission

## Data Availability

The public data sources used in this study, including RNA-seq data from 76 cases (20 ACC: PRJNA601423, 40 MECA: SRP109264, and 16 SDC: SRP096726), were downloaded from the Sequence Read Archive (SRA). Additionally, we uploaded all normal-tumor paired samples derived from all MEC patients in the SRA under PRJNA1014965 accession number. Detailed clinical information is described on Supplementary Table 1.

## Code availability

The reproducible codes used in this study are available on a GitHub repository at https://github.com/pigyun906/SGC.
