# Supplementary material for: Immunological subtyping of salivary gland cancer identifies histological origin-specific tumor immune microenvironment
Source: NPJ Precis Oncol. 2024 Jan 20;8:15. doi: 10.1038/s41698-024-00501-4 (PMC10799913; doi:10.1038/s41698-024-00501-4)
Supplement: Supplementary file 1 — REPORTING SUMMARY [file 41698_2024_501_MOESM1_ESM.pdf]

nature portfolio

Corresponding author(s): Sangwoo Kim

Last updated by author(s): Nov 7, 2023

Reporting Summary

Nature Portfolio wishes to improve the reproducibility of the work that we publish. This form provides structure for consistency and transparency in reporting. For further information on Nature Portfolio policies, see our Editorial Policies and the Editorial Policy Checklist.

Statistics

For all statistical analyses, confirm that the following items are present in the figure legend, table legend, main text, or Methods section.

n/a	Confirmed	
☐	☒	The exact sample size (*n*) for each experimental group/condition, given as a discrete number and unit of measurement
☒	☐	A statement on whether measurements were taken from distinct samples or whether the same sample was measured repeatedly
☐	☒	The statistical test(s) used AND whether they are one- or two-sided<br>*Only common tests should be described solely by name; describe more complex techniques in the Methods section.*
☒	☐	A description of all covariates tested
☐	☒	A description of any assumptions or corrections, such as tests of normality and adjustment for multiple comparisons
☐	☒	A full description of the statistical parameters including central tendency (e.g. means) or other basic estimates (e.g. regression coefficient) AND variation (e.g. standard deviation) or associated estimates of uncertainty (e.g. confidence intervals)
☐	☒	For null hypothesis testing, the test statistic (e.g. *F*, *t*, *r*) with confidence intervals, effect sizes, degrees of freedom and *P* value noted<br>*Give P values as exact values whenever suitable.*
☒	☐	For Bayesian analysis, information on the choice of priors and Markov chain Monte Carlo settings
☐	☒	For hierarchical and complex designs, identification of the appropriate level for tests and full reporting of outcomes
☐	☒	Estimates of effect sizes (e.g. Cohen's *d*, Pearson's *r*), indicating how they were calculated

Our web collection on statistics for biologists contains articles on many of the points above.

Software and code

Policy information about availability of computer code

Data collection	For data collection, we used SRA-Toolkit to download public datasets in Sequence Read Archive (SRA).
Data analysis	FastQC (v.0.11.9), Trimmomatic (v.0.40), STAR (v.2.7.3a), HTSeq (v.0.11.1), DESeq2 (v.1.26.0), GSVA (v.1.44.5), SVA (v.3.46.0), clusterProfiler (v.3.14.3), msigdbr (v.7.5.1), TCGAbiolinks (v.2.21.3), ESTIMATE (v1.0.13), immunoedeconv (v.2.1.0), MIXCR (v.3.0.13), Immunarch (v.0.6.6), and BayesPrism (v.2.0) were used.<br>The codes used in this study are uploaded in Github (https://github.com/Yonsei-TGIL/SGC).

For manuscripts utilizing custom algorithms or software that are central to the research but not yet described in published literature, software must be made available to editors and reviewers. We strongly encourage code deposition in a community repository (e.g. GitHub). See the Nature Portfolio guidelines for submitting code & software for further information.

Data

Policy information about availability of data

All manuscripts must include a data availability statement. This statement should provide the following information, where applicable:

- Accession codes, unique identifiers, or web links for publicly available datasets
- A description of any restrictions on data availability
- For clinical datasets or third party data, please ensure that the statement adheres to our policy

The public data sources used in this study, including RNA-seq data from 76 cases (20 ACC: PRJNA601423, 40 MECA: SRP109264, and 16 SDC: SRP096726), were

downloaded from the Sequence Read Archive (SRA). Additionally, we uploaded all normal-tumor paired samples derived from all MEC patients in the SRA under PRJNA1014965 accession number. Detailed clinical information is described on Supplementary Table 1.

Research involving human participants, their data, or biological material

Policy information about studies with human participants or human data. See also policy information about sex, gender (identity/presentation), and sexual orientation and race, ethnicity and racism.

Reporting on sex and gender	This study includes both males and females.
Reporting on race, ethnicity, or other socially relevant groupings	N/A
Population characteristics	N/A
Recruitment	From March 2012 to April 2018, twenty patients diagnosed with MEC underwent treatment at the Yonsei Head and Neck Cancer Center.
Ethics oversight	N/A

Note that full information on the approval of the study protocol must also be provided in the manuscript.

Field-specific reporting

Please select the one below that is the best fit for your research. If you are not sure, read the appropriate sections before making your selection.

☒ Life sciences ☐ Behavioural & social sciences ☐ Ecological, evolutionary & environmental sciences

For a reference copy of the document with all sections, see nature.com/documents/nr-reporting-summary-flat.pdf

Life sciences study design

All studies must disclose on these points even when the disclosure is negative.

Sample size	We collected all publicly available datasets of SGC and included 20 additional MEC patients.
Data exclusions	In this study, only the primary site was considered if a patient had both primary and metastatic sites.
Replication	N/A
Randomization	N/A
Blinding	In IHC, the fields were chosen randomly by a head and neck/salivary pathologist blinded to the immune infiltration score (IIS) and T-cell infiltration score (TIS) values, in the central region of the tumor.

Reporting for specific materials, systems and methods

We require information from authors about some types of materials, experimental systems and methods used in many studies. Here, indicate whether each material, system or method listed is relevant to your study. If you are not sure if a list item applies to your research, read the appropriate section before selecting a response.

Materials & experimental systems

n/a	Involved in the study	
☐	☒	Antibodies
☒	☐	Eukaryotic cell lines
☒	☐	Palaeontology and archaeology
☒	☐	Animals and other organisms
☒	☐	Clinical data
☒	☐	Dual use research of concern
☒	☐	Plants

Methods

n/a	Involved in the study	
☒	☐	ChIP-seq
☒	☐	Flow cytometry
☒	☐	MRI-based neuroimaging

Antibodies

Antibodies used	Anti-CD45 (DAKO, catalog no. M0701, 0.5 mg/mL) and anti-CD3 (DAKO, catalog no. A0452, 1.2 mg/mL) antibodies were applied and sections were incubated for 24 hours, followed by a 60-minute incubation with biotinylated horse anti-mouse IgG (Vector Laboratories, catalog no. PK-6200) for CD45 antibody, and horse anti-rabbit IgG (Vector Laboratories, catalog no. PK-6200) for CD3.
Validation	N/A

Plants

Seed stocks	N/A
Novel plant genotypes	N/A
Authentication	N/A
