# Supplementary material for: Immunological subtyping of salivary gland cancer identifies histological origin-specific tumor immune microenvironment
Source: NPJ Precis Oncol. 2024 Jan 20;8:15. doi: 10.1038/s41698-024-00501-4 (PMC10799913; doi:10.1038/s41698-024-00501-4)
Supplement: Supplementary file 2 — supplementary information - final submission [file 41698_2024_501_MOESM2_ESM.pdf]

Supplementary Figures

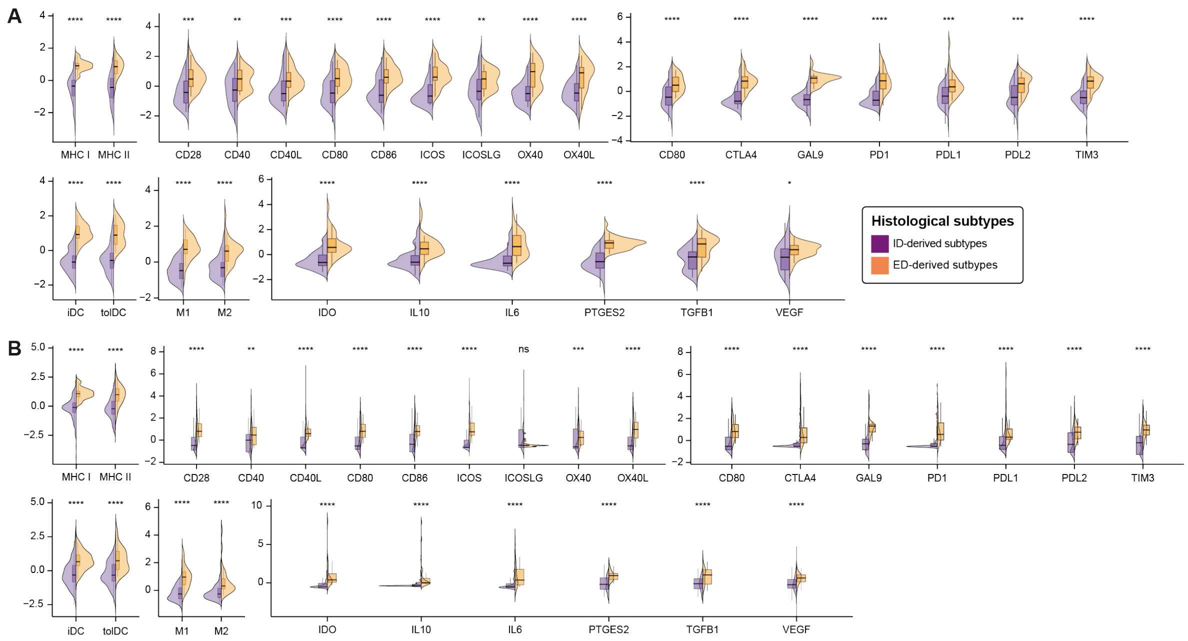

Supplementary figure 1. Comparison of immune-related features between the ID- and ED-derived subtypes. (A) The upper panel displays the results with outlier samples excluded. **(B)** In the lower panel, the same molecules were validated with additional datasets after removing outlier samples.

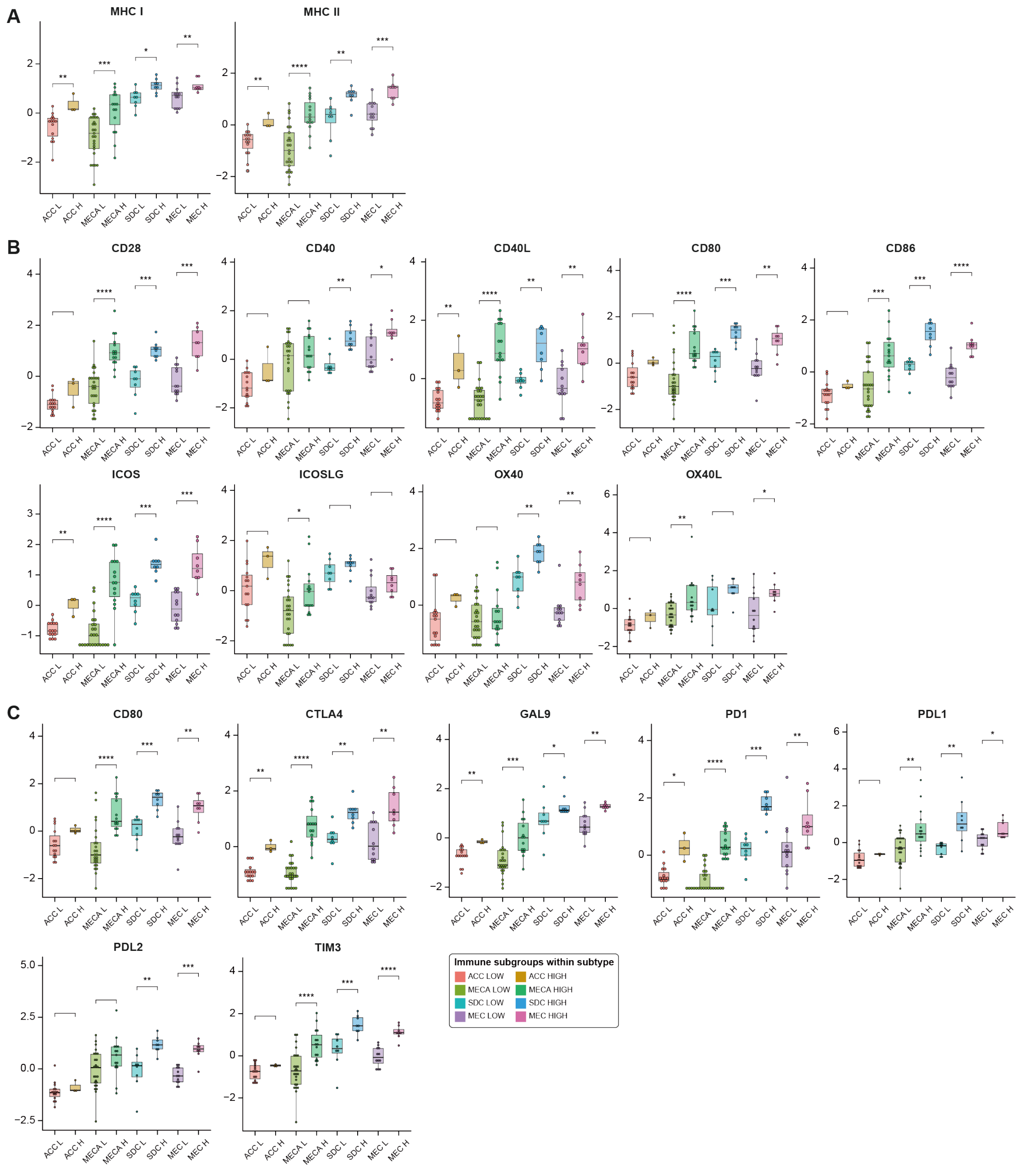

Supplementary Figure 2. Immune subtypes based on the composition of immune cells within each subtype. There are differences in **(A)** MHC molecules, **(B)** immune signaling molecules, and **(C)** immune suppressive molecules between the immune subtypes within each subtype.

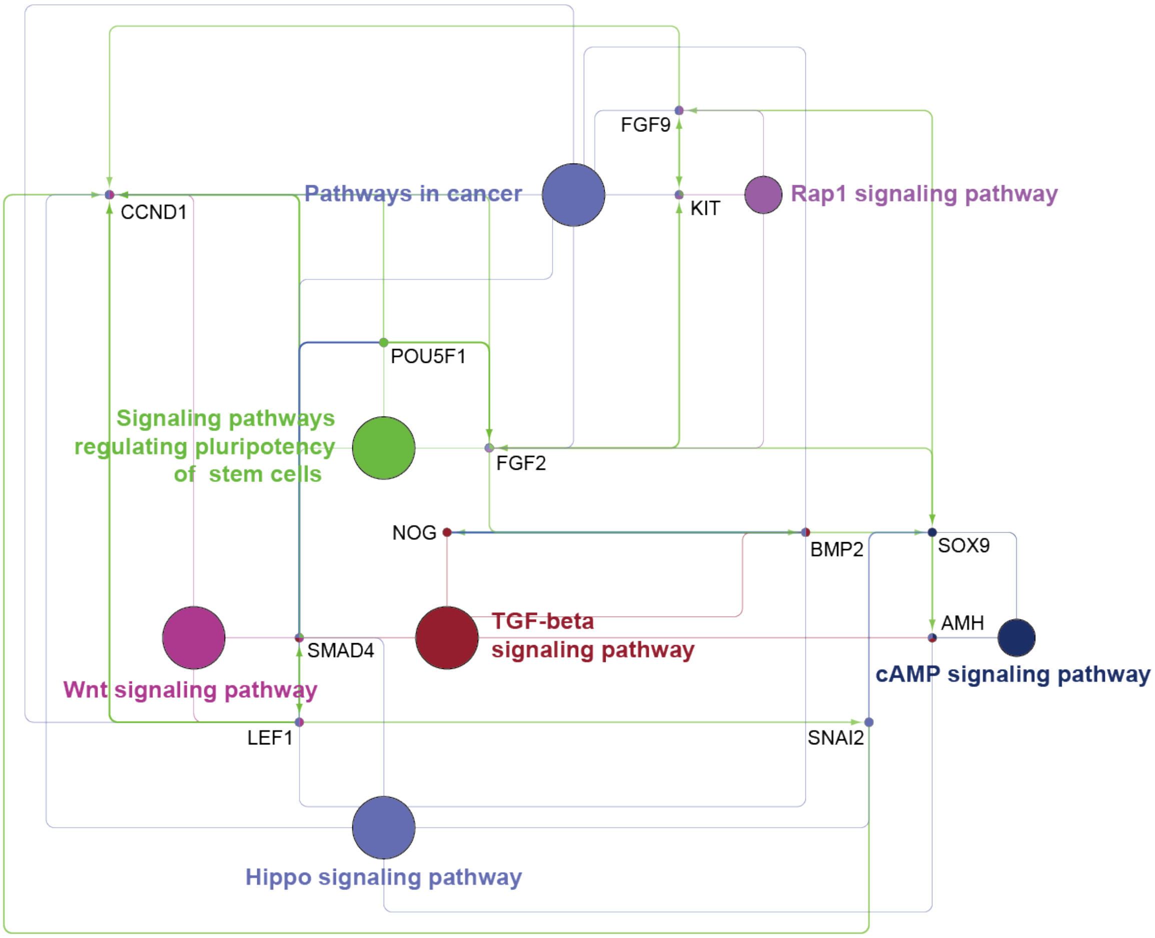

Supplementary Figure 3. Network analysis of up-regulated DEGs in ID-derived subtypes. Green edges and arrows represent activation interaction while blue edges binding interaction between two proteins.

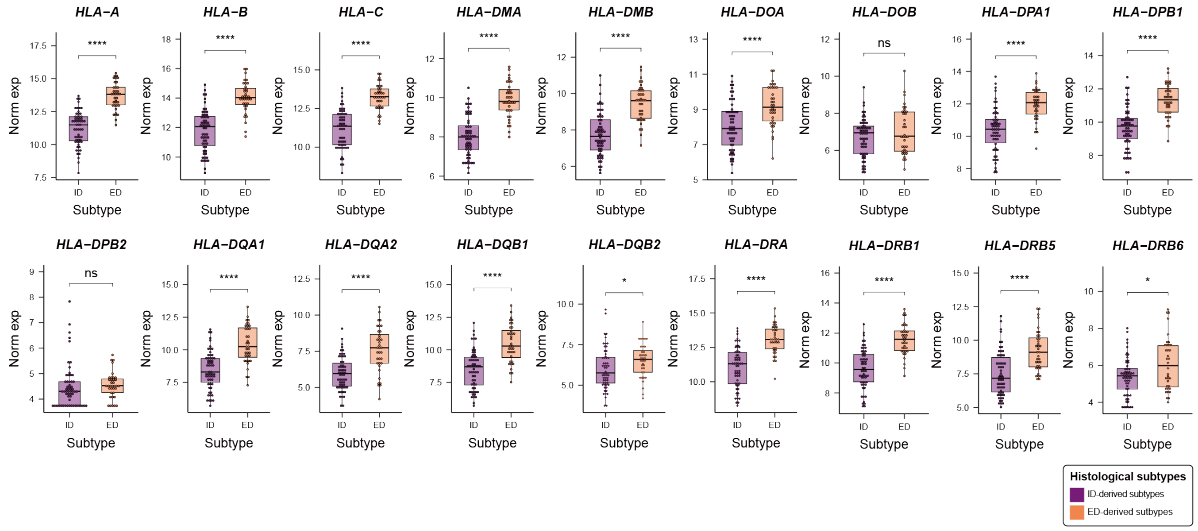

Supplementary Figure 4. Comparison of MHC molecule expression levels between ID-derived and ED-derived subtypes. Normalized expression within each molecule was used for statistical analysis, performed using the Wilcoxon Rank Sum test.

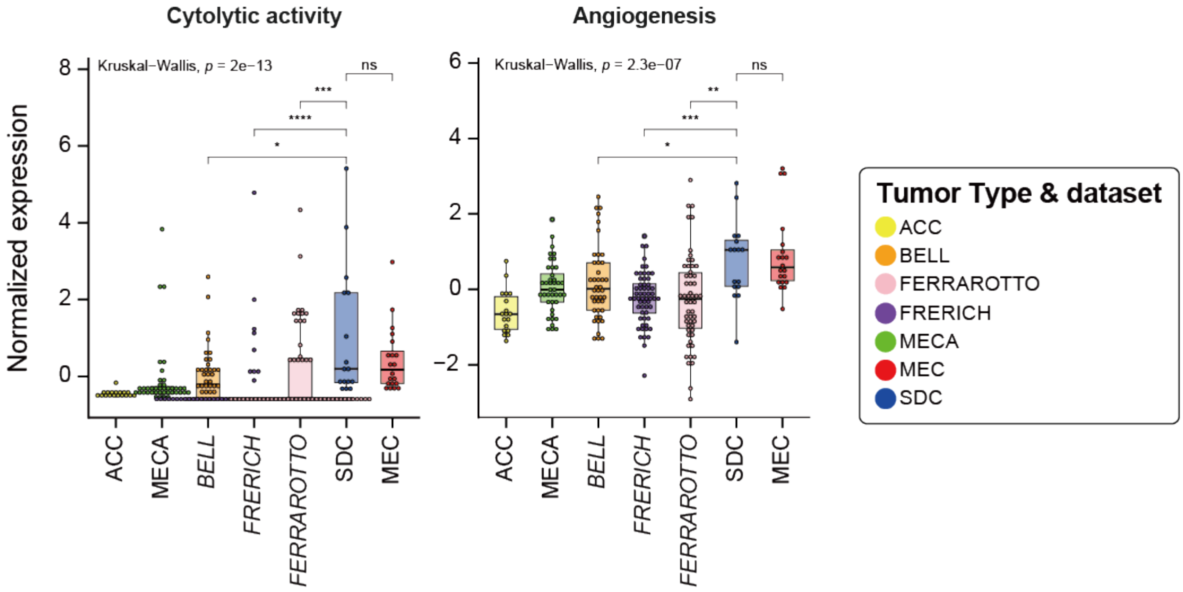

Supplementary Figure 5. Comparison of computationally measured tumor microenvironment scores among histologic subtypes with three independent datasets.

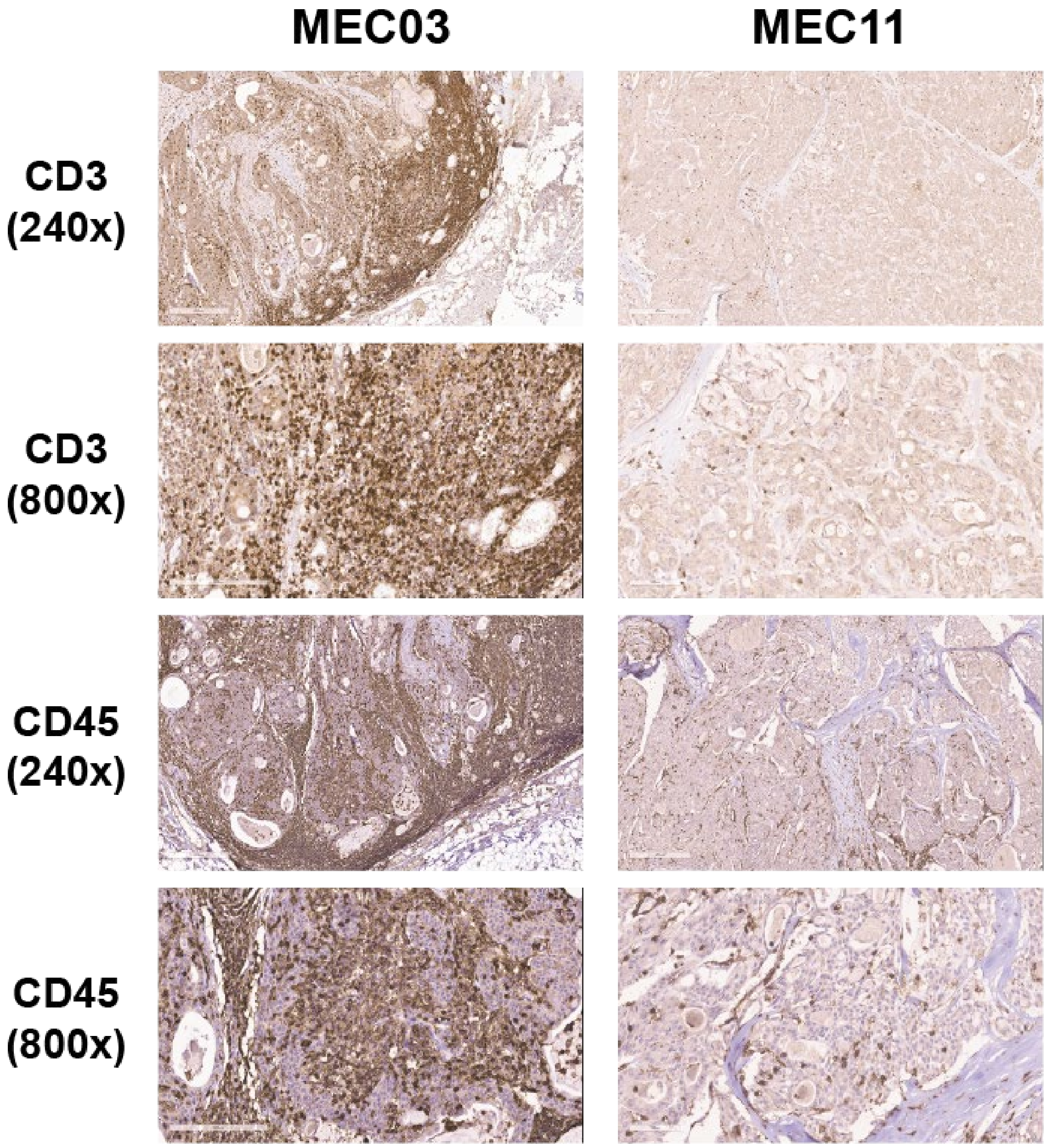

Supplementary Figure 6. Two representative slides of CD3/CD45 IHC in MEC patients.

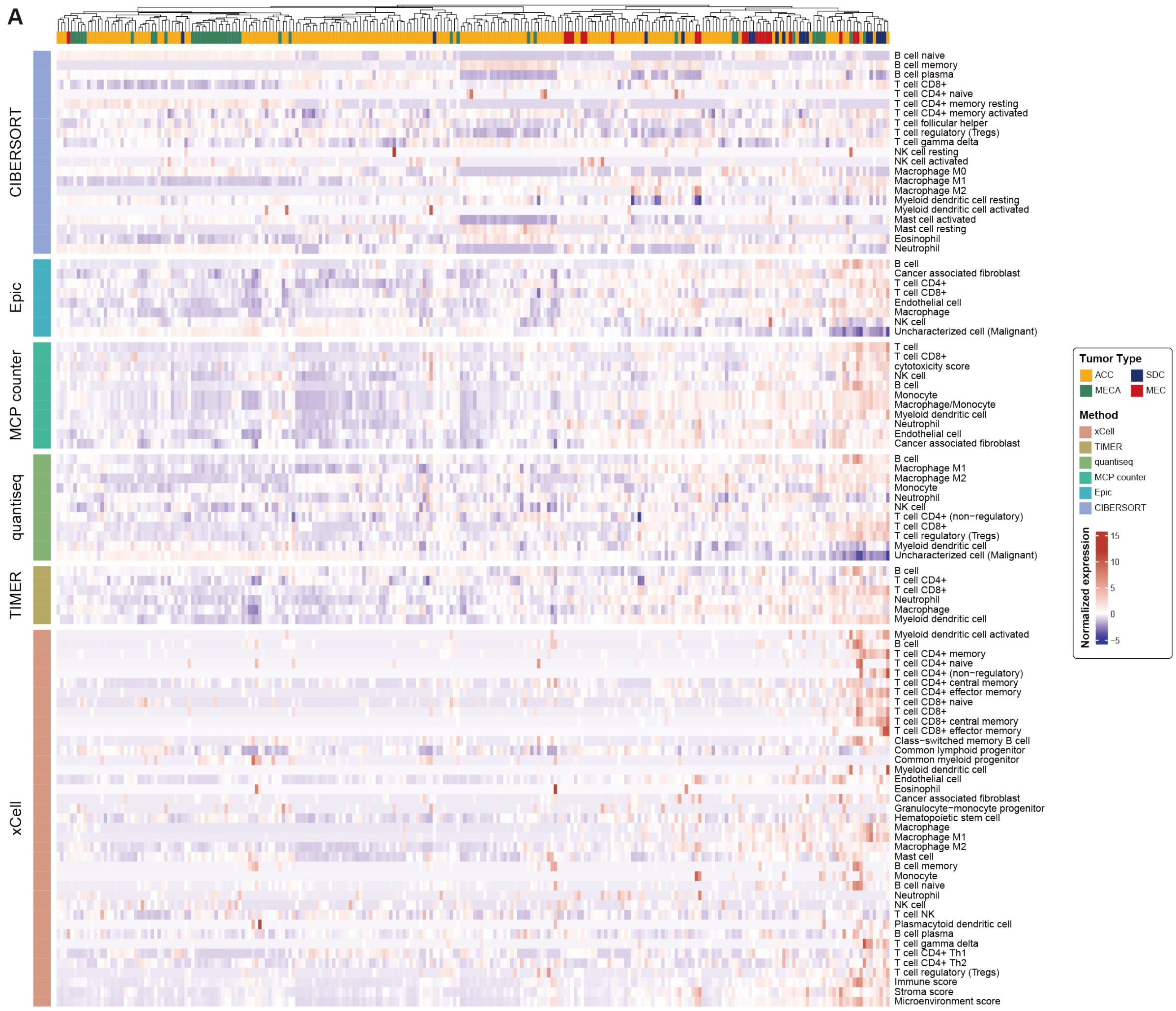

Supplementary Figure 7. Deconvolution and unsupervised clustering based on all immune cell types and scores.

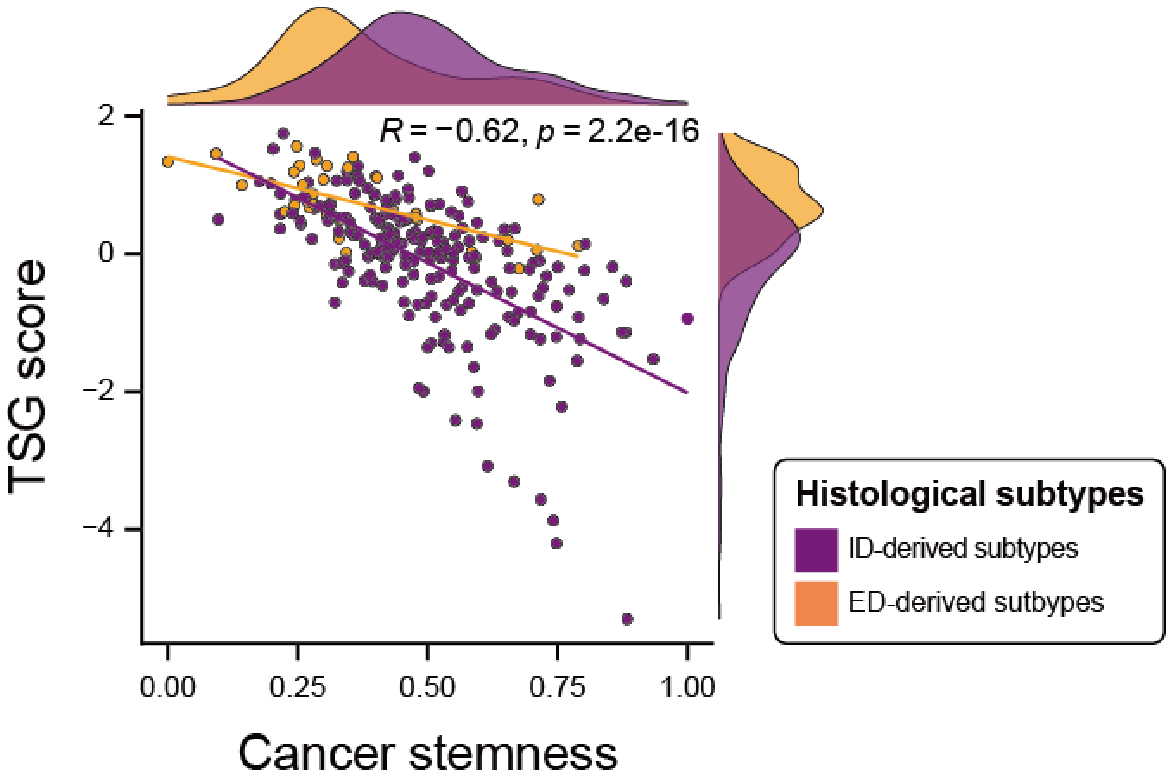

Supplementary Figure 8. Negative correlation between cancer stemness and tumor suppressor gene scores irrespective of histological origins.

Supplementary Tables

Patients ID	Age	Sex	Origin	Grade	Nerve invasion	Node involvement	Disease status	Last f/u	Adjuvant RTx	Description
**MEC01**	33	M	PG	L	O		NED	2013-03-26		
**MEC02**	28	M	PG	L	O		NED	2023-02-06		
**MEC03**	30	F	PG	L			NED	2022-03-28		
**MEC04**	64	F	PG	H		O	NED	2018-06-08		
**MEC05**	63	M	PG	L			NED	2022-11-07		
**MEC06**	31	M	PG	L		O	NED	2019-05-13	O	Postop RT
**MEC07**	51	M	PG	L	O		NED	2019-11-18	O	Postop RT
**MEC08**	70	F	PG	L			NED	2015-08-05		
**MEC09**	18	M	SLG	L			NED	2023-08-18		
**MEC10**	79	M	PG	H	O		DOD	2019-05-23		Nodal recur, DOD
**MEC11**	55	F	SMG	L			NED	2019-11-11		
**MEC12**	80	F	PG	L			NED	2021-02-17		
**MEC13**	48	F	SMG	H			NED	2023-03-15		
**MEC14**	55	F	MS	L			NED	2023-02-22		
**MEC15**	66	F	MS	L		O	DOD	2018-02-01	O	DOD
**MEC16**	24	F	MS	L	O		NA	2022-12-05	O	Postop RT
**MEC17**	58	F	PG	L	O		NED	2023-01-17	O	Postop RT
**MEC18**	52	M	PG	L	O		NED	2023-08-10	O	Postop RT
**MEC19**	47	M	PG	L			NED	2023-04-20	O	Postop CCRT
**MEC20**	67	M	OP-BOT	L			NED	2020-11-26		

Supplementary Table 1. Summary of clinical and pathological information for twenty MEC patients at severance hospital. Abbreviations; MEC: mucoepidermoid carcinoma; M: male; F: female; PG: parotid gland; SLG: sublingual gland; SMG: submandibular gland; MS: minor salivary gland; L: low; H: high; NED: no evidence of disease; DOD: died of disease; Postop RT: postoperative radiotherapy; Postop CCRT: postoperative concurrent chemoradiotherapy.

Signatures	Description
TIME	Comprehensive gene signatures related to immune cell types and scores in the tumor immune microenvironment (TIME) activities [1].
MP	Gene signatures for classifying the polarization of macrophages (MP); M1 and M2 [2]
DC	Gene signatures for classifying subtypes of dendritic cell (DC); pDC and iDC [1].<br>Tolerogenic dendritic cell (tolDC) are estimated using *CD14*, *IL10*, *CCL2*, and *CCL22* [3-5].
SG	Gene expression characterizing major cell types of salivary gland (SG) are utilized from previous study [6].
TSG	1217 human tumor suppressor genes (TSG) were downloaded from the TSGene database [7] and the TSG score was calculated using ssGSEA.
T cell activation	All type of T cell activation marker is collected from various references [8-14].

Supplementary Table 2. Definition of gene signatures and biomarkers used for measuring distinct scores. Abbreviations; pDC: plasmacytoid dendritic cell, iDC: immature dendritic cell, tolDC: tolerogenic dendritic cell,

**Sample**	CD3	CD3	CD3	**CD3 average**	CD45	CD45	CD45	**CD45 average**
MEC1	629	650	729	505.7	702	710	823	663.6
MEC2	67	88	103	54	314	320	323	271.3
MEC3	360	383	523	295.9	833	860	871	703.5
MEC4	131	145	151	92.8	46	51	55	28.6
MEC5	161	163	287	162.4	48	51	67	35
MEC6	188	231	250	164.6	269	312	380	245.6
MEC7	101	85	74	101	244	267	279	216.8
MEC8	71	85	89	41.5	171	189	231	132
MEC9	151	178	213	105	313	376	381	240.2
MEC10	158	213	234	111.9	258	261	277	182.1
MEC11	101	121	133	59.9	133	141	236	86.9
MEC12	103	131	145	79.1	458	617	734	385.2
MEC13	313	442	488	232.6	503	531	568	355.5
MEC14	256	288	312	152.2	267	313	346	190.6
MEC15	156	161	211	105	166	191	201	118.9
MEC16	281	313	365	231.9	271	306	388	217.3
MEC17	213	266	267	159.8	346	414	488	287.3
MEC18	644	670	713	558.2	856	857	870	768.2
MEC19	58	77	78	40.9	147	150	150	110.3
MEC20	91	102	111	69.4	152	166	167	132.4

Supplementary Table 3. Multiplex IHC quantification and cell counts for CD3 (averaged from 3 measurements) and CD45 (averaged from 3 measurements).

Single cell annotation reference							
Ductal/basal epithelial cells	KRT15	SOX2					
Fibroblasts	DCN	LUM					
Duct cells	100A2	WFDC2					
Lonocytes	CFTR	FOXI1					
Mucous acinis	MUC5B	BPIFB2					
Pericytes	MYO1B						
Serous acini cells	LPO	ODAM					
T/NK cells	GZMA	HCST					
Myeloid cells	AIF1	CD163	LYZ				
Muscle satellite cells	PAX7	CD82	NCAM1	MYF5			
Mast cells	MS4A2	TPSB2	GATA2				
Skeletal muscle cells	ACTA1	NEB	MYL2				
Lymphatic endothelial cells	PDPN	PROX1	LYVE1				
Schwann cells	NGFR	SOX10	GAP43	CDH19			
Myoepithelial cells	ACTA2	MYH11	CNN1				
Dendritic cells	CCL13	CCL17	CCL22	CD209	HSD11B1	NPR1	PPFIBP2
Macrophages	APOE	ATG7	BCAT1	CCL7	CD163	CD68	CD84
	KAL1	MARCO	ME1	MS4A4A	MSR1	PCOLCE2	PTGDS
	COL8A2	COLEC12	CTSK	CXCL5	CYBB	DNASE2B	EMP1
	SGMS1	SULT1C2	FDX1	FN1	GM2A	GPC4	SCG5
	CHI3L1	CHIT1	RAI14	SCARB2	CLEC5A		

Supplementary Table 4. Marker for cell type annotation in salivary gland.

Supplementary References

1. Senbabaoglu, Y., et al., *Tumor immune microenvironment characterization in clear cell renal cell carcinoma identifies prognostic and immunotherapeutically relevant messenger RNA signatures.* Genome Biol, 2016. **17**(1): p. 231.
2. Oshi, M., et al., *Abundance of Regulatory T Cell (Treg) as a Predictive Biomarker for Neoadjuvant Chemotherapy in Triple-Negative Breast Cancer.* Cancers (Basel), 2020. **12**(10).
3. Castiello, L., et al., *Expression of CD14, IL10, and Tolerogenic Signature in Dendritic Cells Inversely Correlate with Clinical and Immunologic Response to TARP Vaccination in Prostate Cancer Patients.* Clin Cancer Res, 2017. **23**(13): p. 3352-3364.
4. Layseca-Espinosa, E., et al., *CCL22-producing CD8alpha- myeloid dendritic cells mediate regulatory T cell recruitment in response to G-CSF treatment.* J Immunol, 2013. **191**(5): p. 2266-72.
5. Maldonado, R.A. and U.H. von Andrian, *How tolerogenic dendritic cells induce regulatory T cells.* Adv Immunol, 2010. **108**: p. 111-65.
6. Lin, Q., et al., *Single-cell transcriptomic analysis of the tumor ecosystem of adenoid cystic carcinoma.* Front Oncol, 2022. **12**: p. 1063477.
7. Zhao, M., et al., *TSGene 2.0: an updated literature-based knowledgebase for tumor suppressor genes.* Nucleic Acids Res, 2016. **44**(D1): p. D1023-31.
8. Maecker, H.T., J.P. McCoy, and R. Nussenblatt, *Standardizing immunophenotyping for the Human Immunology Project.* Nat Rev Immunol, 2012. **12**(3): p. 191-200.
9. Raphael, I., et al., *T cell subsets and their signature cytokines in autoimmune and inflammatory diseases.* Cytokine, 2015. **74**(1): p. 5-17.
10. Golubovskaya, V. and L. Wu, *Different Subsets of T Cells, Memory, Effector Functions, and CAR-T Immunotherapy.* Cancers (Basel), 2016. **8**(3).
11. Kumar, B.V., T.J. Connors, and D.L. Farber, *Human T Cell Development, Localization, and Function throughout Life.* Immunity, 2018. **48**(2): p. 202-213.
12. Jameson, S.C. and D. Masopust, *Understanding Subset Diversity in T Cell Memory.* Immunity, 2018. **48**(2): p. 214-226.
13. van den Broek, T., J.A.M. Borghans, and F. van Wijk, *The full spectrum of human naive T cells.* Nat Rev Immunol, 2018. **18**(6): p. 363-373.
14. Mousset, C.M., et al., *Comprehensive Phenotyping of T Cells Using Flow Cytometry.* Cytometry A, 2019. **95**(6): p. 647-654.
